# The new microbiome on the block: challenges and opportunities of using human tumor sequencing data to study microbes

**DOI:** 10.1038/s41592-025-02807-y

**Published:** 2025-09-15

**Authors:** Yingjie Li, Anjun Ma, Evan Johnson, Charis Eng, Subhajyoti De, Sizun Jiang, Zihai Li, Daniel Spakowicz, Qin Ma

**Affiliations:** 1 Department of Biomedical Informatics, College of Medicine, the Ohio State University, Columbus, OH, USA.; 2 Pelotonia Institute for Immuno-Oncology, The Ohio State University Comprehensive Cancer Center – James Cancer Hospital and Solove Research Institute, Columbus, OH, USA.; 3 Division of Infectious Disease, Center for Data Science, Rutgers University, New Jersey Medical School, Newark, NJ, USA.; 4 Department of Genetics and Genome Sciences, School of Medicine, Case Western Reserve University, Cleveland, OH, USA.; 5 Rutgers Cancer Institute of New Jersey, Rutgers, the State University of New Jersey, New Brunswick, NJ, USA.; 6 Center for Virology and Vaccine Research, Beth Israel Deaconess Medical Center, Boston, MA, USA.; 7 Department of Pathology, Dana-Farber Cancer Institute, Boston, MA, USA.; 8 Division of Medical Oncology, Department of Internal Medicine, the Ohio State University Comprehensive Cancer Center, Columbus, OH, USA.; 9 Deceased: Charis Eng.

## Abstract

Microbes within tumors have been recognized and experimentally related to oncogenesis, tumor growth, metastasis and therapeutic responsiveness. Studying the tumor microbiome presents difficulties, as early indications suggest that microbe populations are low in abundance, sparse and highly heterogeneous. Disparate results from computational profiling of the tumor microbiome have cast doubt on the premise of microbes in tumors. Yet decades of experimental evidence support the presence of tumor microbes, at least in a limited number of tumor types. In this Perspective, we discuss the importance of iteratively improving microbe-targeted sequencing techniques, established analytical pipelines, robust computational tools and solid validations to address current challenges and fill existing knowledge gaps. The vast amount of human tumor sequencing data available could greatly enhance systematic investigations of microbiome–tumor interactions with methods to quantify the composition of the tumor microbiome accurately.

Microbes are involved in many aspects of cancer, including direct and indirect impacts on oncogenesis, progression, metastasis and treatment responses^1–3^. While much previous research has centered on gut microbes due to their abundance and accessibility^4,5^, tumor microbes have gained increasing attention for their tumor-specific roles and local interactions within the tumor microenvironment (TME)^2,6–9^. Although the source of the microbes and factors affecting their immigration and extinction rates remain largely unknown, understanding which microbes contribute to tumor progression and how they communicate with antitumor immunity has the potential to impact cancer prediction and therapy^10^.

The year 2020 marked a pivotal moment for tumor microbiome research. Poore et al. used human sequencing data to identify microbiome reads in 33 cancer types in both tumor tissues and blood samples from whole-genome sequencing data^11^. This was the first study describing cancer type-specific microbiome signatures from host sequencing data and the diagnostic value of circulating microbial DNA. This work attracted immediate attention, leading to studies adopting the same analytical approaches or directly using their results^12–17^. However, a critical reevaluation study by Giwahi et al. criticized the methods and conclusions, arguing that observed microbes were mostly false positives^18^. Furthermore, they showed how the methods could lead to technical artifacts that drove those provocative cancer type-specific microbiome signatures. Much of the discourse occurred publicly on social media and was summarized by *the New York Times*^19,20^. In June 2024, the original 2020 paper by Poore et al. was retracted^21^. The retraction underscores the importance of rigorous data reanalysis, the proper use of existing tools to avoid significant biases and the necessity of experimental validation. The complexity of these tools and pipelines must weigh against their potential benefits, and the field requires improved methods for tumor microbiome inference from host sequencing data to advance our understanding of this enigmatic ecosystem.

As terminologies in this field remain inconsistent, it is important to clarify the definitions and relationships. Therefore, we propose standardized definitions and nomenclature based on existing studies to promote precise communication and facilitate broader adoption^22–26^ (Box 1). Specifically, in this Perspective, we use ‘tumor microbiome’ to describe the genomes of microbes in the TME and adjacent surrounding normal tissues measured by nucleic acid sequencing, the primary focus here. To distinguish between different origins of tumor microbes, we use the term ‘tumor-resident (or colonizing) microbes’ to refer to microbes that stably colonize the TME, while defining ‘tumor-infiltrating microbes’ as those that enter solid tumors via the bloodstream but may not be long-term residents of the tumor niche.

Therefore, we believe that now is an opportune time to summarize the current challenges and outline a roadmap for advancing data analysis in tumor microbiome research. In line with this vision, we propose developing microbe-targeted sequencing techniques, rigorous analytical tools and pipelines and solid validation to maximize the effectiveness of tumor microbiome discovery and association studies.

## Evidence for the tumor microbiome

**Fig. 1 |:**
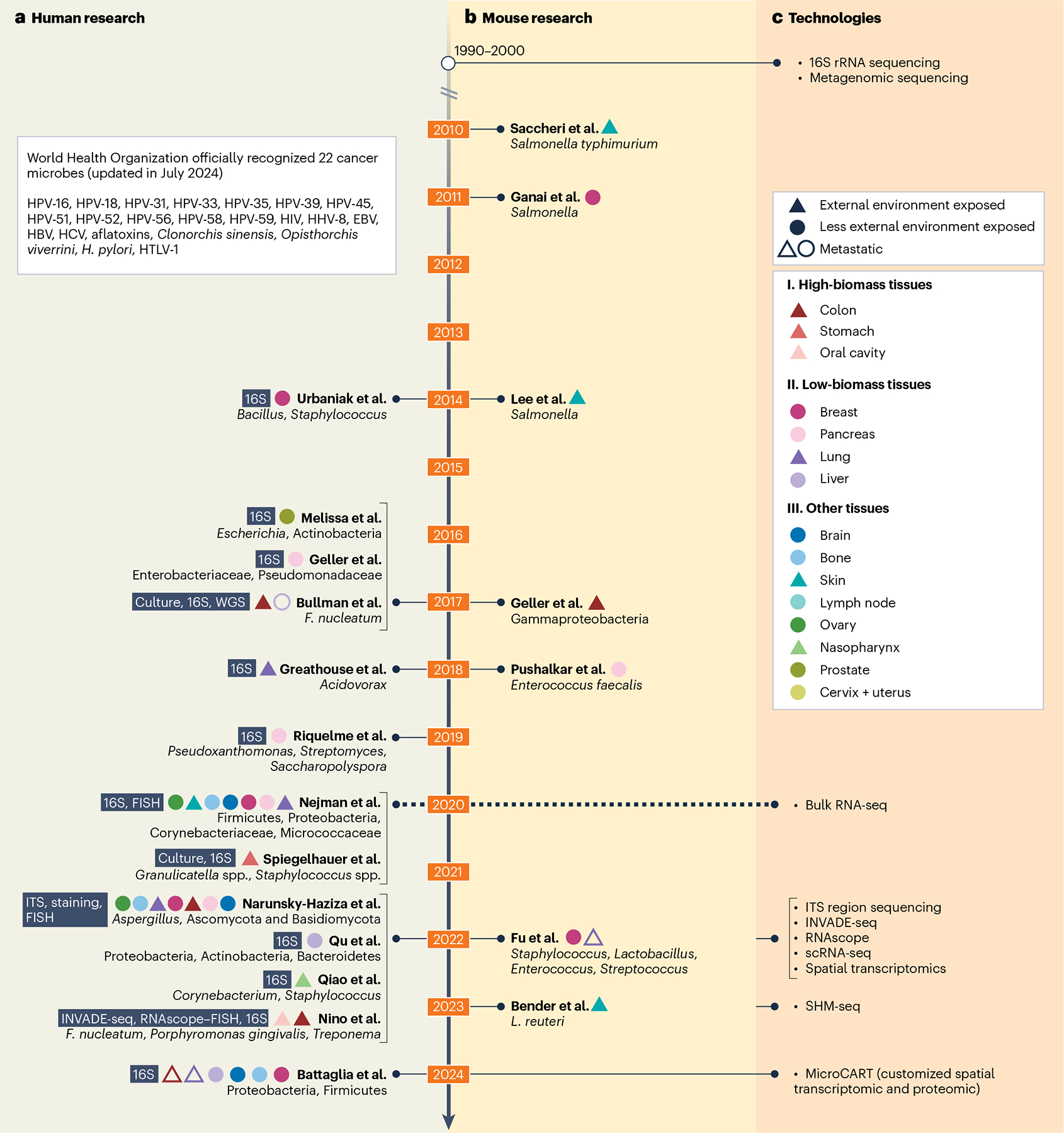
Evidence and technologies emerging for the tumor microbiome. **a**, Timeline of recent tumor microbiome studies in humans, highlighting microbiome existence across different tumor tissues, including both high- and low-biomass tumors. The year 2020 (dashed line) marked a breakthrough in tumor microbiome research, as microbiomes were derived from host-based sequencing data, significantly bringing impact to this field. Circle and triangle nodes in different colors represent tumor tissues biopsied from various organs: nodes with no fill represent tumor tissues biopsied from metastatic organs, while navy highlighting indicates the detection techniques used in each study. EBV, Epstein–Barr virus; HBV, hepatitis V virus; HCV, hepatitis C virus; HHV, human herpesvirus; HIV, human immunodeficiency virus; HPV, human papillomavirus; HTLV-1, human T lymphotropic virus 1; 16S, 16S rRNA sequencing; FISH, fluorescence in situ hybridization; ITS, internal transcribed spacer; WGS, whole-genome sequencing. **b**, Tumor microbiome studies conducted in mouse models. **c**, Overview of advanced techniques used to study the tumor microbiome. The application of 16S rRNA sequencing and metagenomic sequencing to solid tissues dates back to the early 2000s. In the past 2 years, internal transcribed spacer region sequencing and customized sequencing technologies have been developed to analyze microbiomes at both the single-cell level and the spatial level. Notably, INVADE-seq enables microbiome sequencing at the single-cell level in tumor studies. At the spatial level, SHM-seq and MicroCART have been introduced for customized spatial transcriptomics and proteomics, although their applications in tumor tissues remain limited. scRNA-seq, single-cell RNA-seq.

The current debate has centered on detecting nonhuman nucleic acids in bulk tumor sequencing data. It has led some to question whether all tumor microbiome observations are false positives^18^. However, observational and experimental evidence using several techniques, including non-nucleic acid-based ones, generated well before and after the current debate, has shown the presence of microbes (Fig. 1). The association between 11 microbes and cancer was officially recognized by the World Health Organization in 2009 (ref. 27) (and was updated to 22 microbes in 2024). Most of them are viruses, including Epstein–Barr virus, hepatitis B virus, human papillomavirus and human immunodeficiency virus, and have been widely confirmed in the field^28,29^. Limited but rapidly expanding evidence driven by technology advancement and experimental validation suggests the presence of nonviral microbes in the TME^6–8,30,31^.

The tumor microbiome in humans varies by organ site, often correlating with proximity to external environments (Fig. 1a). Tumors in the alimentary canal, such as the colorectal, gastric and oral cavity, tend to harbor a relatively high microbial burden (Fig. 1, high-biomass tissues). In the past decade, studies have focused on colorectal cancers, the most recognized high-biomass tumor type in the human body, to investigate microbial invasion and local tumor–microbe interactions in the intestine and colonic mucosa^6,8^. For example, Bullman et al. identified *Fusobacterium nucleatum* in colorectal cancer and identified its presence in primary tumors and distant metastases^6,8^. Similarly, *Granulicatella* spp. and *Staphylococcus* spp. were found in gastric cancer^32^, while *Parvimonas* and *Peptoniphilus* were enriched in oral squamous cell carcinomas^8^. By contrast, tumors in tissues traditionally considered ‘sterile’, such as the pancreas, liver, lung and breast, likely have low microbial burden (Fig. 1, low-biomass tissues)^7,31,33–35^. Sequencing-based evidence has also suggested the presence of microbes in brain^7,36^, bone^7,36^, skin^7^, ovary^7,36^, nasopharynx^24^ and prostate^37^ tumors (Fig. 1, other tissues). Support for the plausibility of microbes in diverse tissues can be found in the noncancer literature. Microbes have been observed throughout the body but are quickly cleared by the immune system^38^. Routine oral care leads to transient bacteremia^39^, and microbes have been cultivated from human tissues, such as omental fat^40^. Moreover, recent studies have demonstrated that microbes can translocate from the gut during homeostasis^41^. This suggests that low-abundance microbes enter circulation from various sources, and their persistence is a function of the local environment and their clearance rate. Tumors tend to have chaotic circulation and are immune privileged^42^, which could lead to microbe deposition and longer persistence. These findings could explain the presence of microbes in tumors of tissues previously thought to be free of microbes.

An additional layer of credibility is added by experimental mouse studies (Fig. 1b). These studies often rely on the delivery of microbes by oral gavage. For example, in 1979, Berg and Garlington cultured microbes from mouse lymph nodes over 100 d after oral gavage of the same bacteria^43^. Riquelme et al. used mouse models to confirm the immune-modulating effects of microbes observed in human pancreatic tumors^33^. Bender et al. observed oral gavage-delivered *Lactobacillus* in orthotopic melanoma tumors^44^. In addition to direct delivery, some studies have demonstrated tumor microbiome transmission. Bullman et al. found *Fusobacterium* in patient-derived xenograft mouse model tumors that traveled with metastases^6^. Pushalkar et al. found that intestinal bacteria migrated into the pancreas^45^. Gammaproteobacteria^46^, *Salmonella*^9,47–49^, *Escherichia*^50^, *Clostridium*^51^ and *Bifidobacterium*^52^ are enriched in tumors as a function of chemotaxis properties (for example, the trace amine-associated receptor detects aspartate secreted by viable cancer cells and the trace amine-associated receptor promotes migration toward ribose in necrotic tissue^53^ and oxygen tolerance). In addition to mice, other animal models, such as rhesus monkeys, have also been used to study the tumor microbiome^54,55^. The accumulation of microbes in tumors and the experimental manipulation of the tumor microbiome open the door to future translation to therapeutic use, such as directing chimeric antigen receptor T cells to solid tumors using an oral probiotic^56^ or tail vein injection. Nevertheless, these models are limited in addressing the ‘natural’ colonization of microbes in human tumors. They often are syngeneic heterotopic, injected tumors colonized with atypical routes (for example, high-dose injection into the tail vein). Some effects in mice were not reproduced in humans, such as when researchers injected attenuated *Salmonella* into patients with melanoma. Some tumor colonization occurred at the maximum tolerated dose but no regression, despite similar microbe concentrations leading to smaller tumors in mice^57^. Despite these shortcomings, we believe that the weight of the presented evidence justifies the continued efforts to search for tumor microbes using high-throughput sequencing data.

Technological advances are improving the ability to make complex inferences in human samples. The rapid progress in tumor microbiome research has driven technological advancements, which, in turn, have further accelerated research in this field (Fig. 1c). Notably, 16S ribosomal RNA (rRNA) sequencing, beginning in the 1990s, remains the dominant method of detecting bacteria due to the sensitivity of ribosomal gene amplification. The application of metagenomic sequencing in the 2000s was often a byproduct of whole-genome shotgun sequencing methods to identify somatic mutations^58^. Internal transcribed spacer sequencing, developed in the 1990s and targeting a variable region on fungal ribosomes, was documented as being applied to tumor tissues in 2022 (ref. 36). There are well-documented challenges with amplicon-based methods in cancer and other fields^59^ that should not be ignored, including when prominent results have been called into question^60^. Culturing remains a common method that confirms the presence of a live organism^33,44^, and many in the field are pushing for this to return to prominence as the standard validation technique. However, many microbes remain difficult to culture in the laboratory. Microscopy-based methods have been used with antibody-based observation of bacterial lipopolysaccharide, lipoteichoic acid or oligonucleotide hybridization to 16S or other genes^7,36,61^. Newer fluorescence-based methods, such as RNAscope, have shown particular utility in this context, in which the multiple-oligonucleotide-matching requirement adds specificity and multiple-fluorophore signal amplification adds sensitivity^8^.

The year 2020 was a watershed moment in the tumor microbiome research field. Since the release of Poore et al.’s paper^11^, papers describing the tumor microbiome have tended to have three distinct qualities from pre-2020 papers that broadly reflect advances in computation and sequencing technologies (Fig. 1, above versus below the dashed line). (1) More pan-cancer studies. For example, Nejman et al. comprehensively analyzed over 1,500 tumor samples, including adjacent normal regions, across seven cancers by using 16S rRNA sequencing. This study also confirmed bacterial presence through immunohistochemistry anti-lipopolysaccharide antibodies and fluorescent d-alanine labeling of intracellular live bacteria^7^. (2) Integration of bulk tissue sequencing with other sequencing modalities, such as single-cell RNA sequencing (RNA-seq). The relatively low microbial biomass in tumors has led to the development of advanced techniques that integrate customized microbial probes to enable simultaneous host and microbial signal extraction^8,62^. For example, invasion–adhesion-directed expression sequencing (INVADE-seq) allows host single-cell transcriptomic analysis with targeted primers in single-cell RNA-seq, facilitating the identification of microbe-associated human cells^8^. Some studies have emphasized integrating multiple methodologies and including rigorous in vitro validation experiments^7,8,32^. (3) Integration of spatial approaches. Identifying the tumor microbiome from spatial transcriptomics data enables the visualization of microbial RNA within host cells or tumor niches, thereby providing a ‘spatial signature’ defining the interaction of microbes and the TME^61^. Techniques such as spatial host–microbiome sequencing (SHM-seq)^63^, microbiome cartography (MicroCART) for customized GeoMx Digital Spatial Profiler spatial transcriptomics or spatial proteomic profiling with 16S-specific oligonucleotide probe designs allow the simultaneous acquisition of host- and microbial-specific antibody-derived signals or 16S sequences, respectively^64^. Recent adaptations of in situ polyadenylation to enable poly(T) capture of microbial sequences (which generally do not have a poly(A) tail) with spatial sequencing approaches also promise to unravel host–microbial interactions within the tissue microenvironment^65^.

Given the above evidence and its limitations, we believe that the following challenges need to be solved to advance tumor microbiome research, particularly using bulk high-throughput sequencing data.

## Challenge 1: tumor microbiome research requires a large amount of FAIR (findability, accessibility, interoperability and reuse) data

**Fig. 2 |:**
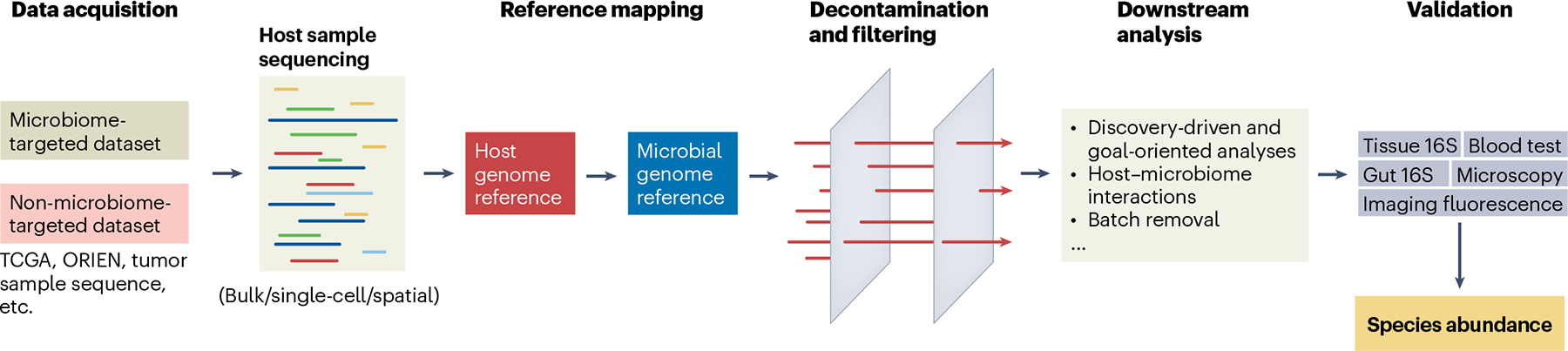
A general and typical computational framework for host tumor microbiome analysis. Steps include data acquisition, reference mapping, decontamination and filtering, downstream analysis and prediction validation.

The study of the tumor microbiome requires large datasets, challenging the current sharing standards (Fig. 2). Microbes have been identified in many tumor sequencing data types generated by strand synthesis, including bulk, single-cell and spatial sequencing technologies. DNA-sequencing approaches along with total RNA-seq (with ribosomal depletion) are effective ways of extracting both host and microbiome profiles from tissues. In addition, specific protocols have been employed to enrich microbial reads for all or targeted sets of microbiomes^66^. However, microbes can even be found when technical selection reduces the observed counts (for example, poly(A)-selected RNA-seq). Even the most stringent computational pipelines mapping to only finished bacterial genomes find microbes in many tumor types^18^. However, the data also indicate that most, if not all, species are uncommon and present in small quantities (low prevalence and abundance); therefore, datasets with even hundreds of tumors are often under-powered. Furthermore, early analyses of multiple datasets or diverse cohorts (for example, the Cancer Genome Atlas Program (TCGA), the Oncology Research Information Exchange Network (ORIEN) and the Black Women’s Health Study) suggest many differences in microbial populations^67,68^. The differences may be due to the demographics of patients, time of collection, geography or technical features when sample collection is uneven across clinical centers. To avoid center-specific contaminants confounding microbiome signals, a proper study design would involve collecting relatively balanced numbers of samples for each tumor type across multiple centers. Yet, most of the available retrospective host sequencing data, although massive, are not specially designed for microbiome capture and detection, including TCGA and ORIEN, and robust and reliable computational attempts are needed to fill the gap (Fig. 2).

On the other hand, it is noteworthy that, for the utility of multiple datasets to infer microbial-associated results, researchers should also consider variation between technical platforms and samples. Several microbiome-specific tools have been developed to remove batch effects, thereby ensuring more reliable data integration across studies^69–71^. Finally, those datasets must be shared in a way that challenges paradigms regarding protected health information. Efforts toward accessing such datasets while avoiding privacy leakage led to the generation of pBAM, that is, a privacy-preserving BAM format^72^. In addition, processing the data to find microbes requires raw, often protected data. Still, there are mechanisms to accommodate this issue; controlled access to repositories (for example, dbGaP^73^) is a rigorous way to protect confidentiality while providing access. Unfortunately, there is a growing trend in which raw files of studies are not submitted upon publication. When the National Institutes of Health (NIH) funded most sequencing efforts, it was reasonable to require that all data be made available to researchers. Now, a common business model (for example, Aster Insights or Tempus) is to sequence many tumors and then sell access to bespoke analyses of that cohort. While researchers may benefit from access to the data, the business model depends on maintaining privileged access. They partner with researchers but do not release the raw data upon publication. This market is expected to grow, and it challenges the current paradigm because other researchers cannot regenerate the microbe dataset from scratch or reprocess the data with a different pipeline to compare the results. Furthermore, current data standards do not include access to unmapped human reads. Depositing metagenomic data into the Sequence Read Archive repository automatically scrubs human reads from all metagenomic data^74^; the same process for tumor sequencing data could be used to reduce the need to access raw data. At the very least, researchers should process publicly available samples, standardized synthetic datasets, spike-ins and/or gold-standard datasets so that other researchers can compare the results of different pipelines without accessing the raw data.

## Challenge 2: false positives and false negatives pervade tumor microbiome analyses

False positives in deriving the tumor microbiome can originate from several sources, including contamination during sample collection, preservation, extraction, library generation, sequencing and processing^75,76^ (Table 1). Efforts to reduce these effects are complicated by the previously described need for large-scale datasets, which often lack negative controls. Nejman et al. processed over 800 blank negative controls alongside actual samples to decontaminate the data^7^. The use of extensive negative controls, which did not contain any DNA, allowed for the effective identification and removal of laboratory-borne contaminants, a process not inherent in TCGA datasets. This approach has also been applied in fungal profiling work to enhance the decontamination of TCGA data^36^. The same cohort used for bacterial profiling with 16S rDNA sequencing was employed for fungal analysis with negative controls to generate a decontaminated cohort. Nejman’s team cross-referenced fungal reads in TCGA samples against their own decontaminated cohort and public resources, such as the Human Microbiome Project’s gut mycobiome cohort. Although host sequencing datasets such as TCGA originally lacked these negative controls, such an approach significantly improved the quality of fungal data by facilitating the identification and removal of contaminants. As we continue to leverage genomic data in future studies, including negative controls can greatly enhance our ability to accurately identify and analyze nonhuman reads. Some researchers use statistical methods to infer and mitigate potential contamination: (1) identifying significant negative associations between microbe abundances and the concentration of the input nucleic acid (the lower the input, the higher the microbe abundance, suggesting that it was present at a fixed concentration on purification columns or in buffers^77^), (2) analyzing paired samples expected to have fewer microbes (for example, blood)^12^, (3) filtering to microbes validated by an alternative method (for example, 16S rRNA sequencing)^78^ and (4) applying the relatively crude but effective strategies of removing taxa commonly found in negative controls from other contexts^75^.

As shown above, much emphasis has been placed on the danger of false positives, which remains a dominant concern. The field needs the release of fully sequenced negative controls from each collection site and processing batch and a dedicated effort to improve the quality of reference genomes. We propose that there is also likely to be a high false negative rate that should be considered when evaluating the field’s promise. Microbes are depleted in most tumor sequencing preparations. Extraction conditions are gentler than those used for microbe-dominant samples, and often there is some selection for regions of interest (for example, human exons, transcripts). Many of the filtering methods, particularly those that exclude a ‘blacklist’ of microbes commonly found in sequenced negative controls, exclude taxa that are likely candidates for surviving in conditions of varying oxygen levels (for example, traversing from the gut to a tumor), leading some researchers to ‘whitelist’ common human commensals. In some cases, certain bacteria (for example, pan-cancer-associated genera like *Fusobacterium*) are frequently included in blacklists and thus must be explicitly whitelisted to be retained in downstream analyses. Adding to the challenge is the newness of the field. The exRNA Human Common Fund Project grappled with similar challenges when finding nonhuman sequences in extracellular vesicle preparations, leading to questions about dietary contributions^79^. Given these challenges, the design of the experimental protocol is equally critical for reducing both false positives and false negatives. We believe that the risk of false positives and negatives should be a function of the dataset, whether it has sequenced negative controls, whether care was taken to ensure sterile extraction, whether cells were handled with sufficient force to capture microbes, etc. On balance, the danger of false positives in datasets like TCGA outweighs the likelihood of false negatives. To that end, robust computational pipelines and/or tools that are tolerant of or can control for false positives and negatives are necessary for any downstream host–microbiome analysis.

## Challenge 3: building a rigorous pipeline for tumor microbiome analysis is not trivial

To provide practical guidance for computational pipeline construction, we summarize bioinformatic approaches in recent tumor microbiome studies (Table 2). All pipelines start with the read alignment step. Classic alignment tools perform pairwise sequence alignment that is extremely slow for large-scale data profiling. For example, BWA is designed for mapping reads to known reference genomes, yet it lacks taxonomic classification capabilities. PathSeq employs a subtraction-based strategy of host removal and filtering and increases processing speed by aligning nonhost reads to microbial genomes using BWA-MEM^80^. By contrast, Kraken (including Kraken2 and KrakenUniq) constructs *k*-mer-based reference pools and uses all genomes rather than focusing only on marker gene regions, which is the most widely used alignment tool for tumor microbiome research. It has also been reported that combining Kraken2 with a deep learning neural network can enhance the taxonomic classification of metagenomic contigs^81^. Meanwhile, the selection of human and microbial reference genomes is critical. The T2T-CHM13 human reference genome, for instance, extends coverage to previously missing regions and the Y chromosome, which are absent from commonly used references such as Hg38 and Hg19. To evaluate the impact of reference genomes on microbial read detection, ablation tests are commonly used to compare the number of remaining microbial reads after aligning to different human reference genomes using the same microbial database or to the same human genome with different microbial reference databases^30,82^. However, some human sequences may persist in the ‘nonhuman’ read set due to no ‘perfect’ complete reference genomes, sequence homology between human and microbial DNA or limitations in alignment stringency. Thus, reference genome cleaning is needed. To address human sequence contamination in the ‘nonhuman’ reads, Gihawi et al. proposed constructing microbial reference databases that include only complete genomes from microbes, human reference genomes and laboratory vectors to reduce false positives^18^. However, this approach has not been systematically evaluated, as no direct comparison has been conducted using the same nonhuman reads processed by both a ‘complete’ reference and an original reference. The ablation tests by Poore et al. to challenge the validity of customized ‘complete’ reference genomes are also indirect, highlighting the need for a well-designed evaluation of these pipelines. A more pernicious problem is the contamination of one organism in the reference genomes of other organisms. Bacterial contamination in other bacterial or fungal genomes and the omnipresence of common laboratory vectors is a relatively well-established concern^18,83^. More challenging in this context is the problem of human contamination in draft bacterial genomes and residual human sequences persisting in the filtered reads after mapping to human and microbial genomes, leading to inflated bacterial counts^84^. Lastly, relying solely on complete bacterial genomes may lead to false negatives, as microbes that exist only in metagenome-assembled or draft genomes would be excluded. To balance the need for comprehensive microbial genome coverage while minimizing human sequence contamination, tools such as Conterminator^85^ and Exhaustive^82^ have been developed to align human reference genomes against microbial databases and remove potential contaminants.

Next, the filtering step is to filter out low-quality reads and remaining contaminants. Commonly used criteria include read count thresholds, genome coverage and *k*-mer abundance when using Kraken-based alignment tools. Some pipelines incorporate statistical tests to remove false positives based on biological features. For example, one approach requires microbes to exhibit a correlation between total reads and unique *k*-mers within a sample^86^. The selection of appropriate thresholds and statistical tests depends on the specific dataset characteristics. Proper benchmarking and validation of identified microbes can help refine filtering metrics and establish more reliable standards. The batch correction step is the final critical aspect, aiming to remove variation generated by technical factors (for example, different platforms and data collection methods). One study argued that microbial signals may be altered after batch correction, raising concerns about potential biases^18^. Tools like ConQuR^71^ were specifically designed to address batch effects in microbiome data, while Voom-SNM^82^, originally developed for host sequencing data, has also been adapted to microbiome data. In the correction step, researchers should also consider removing bias induced by clinical center-specific contaminants^82^, along with a careful study design to ensure proper sample collection.

The challenges and limitations described above lead to the conclusion that solely using one modality (for example, metagenomics, bulk transcriptomics and spatial transcriptomics) is insufficiently rigorous for studying the tumor microbiome. To improve robustness and accuracy, integrating complementary approaches such as cultivation for microbial viability, immunohistochemistry and microscopy, imaging, in vitro assays and animal models or interventional experimental designs remains necessary.

## Perspective: data-driven and hypothesis-driven analyses toward insight discovery in tumor–microbe interactions

A pipeline that controls false positive and false negative signals in the data is foundational to any tumor microbiome study, yet the game just begins^10,87^. Downstream analysis of tumor microbiome research leverages two complementary analytical frameworks to uncover insights into tumor–microbe interactions: data-driven exploration and hypothesis-driven inquiry.

In a data-driven approach, investigators analyze large-scale datasets without preconceived assumptions and use computational methods and multiomic profiling to guide discovery. This unbiased strategy can reveal unexpected associations between microbial communities and tumor characteristics, potentially leading to new hypotheses. For example, data-driven analyses might pose broad questions or lead to subsequent mechanistic investigations such as (1) which microbes can be found in tumors, even rarely; (2) what microbe–host interactions exist in different tumor types, and what do they suggest about potential relationships with cancer progression; (3) how does the composition of the tumor microbiome differ between early-stage and late-stage cancers, and do these differences affect tumor development or metastasis; (4) which microbial genes or metabolic pathways are significantly enriched in tumor-associated microbial communities compared to normal tissues, and what might these functional signatures indicate about microbial contributions to tumor biology; and (5) how do tumor-infiltrating microbes affect local innate and adaptive immune responses, and how does that affect cancer outcomes? One solution, among others, is to develop machine learning methods well suited for efficiently processing sparse data without requiring dense connections between samples or cells and microbes. Several approaches, including MICAH^88^ and MintTea^89^, lay the groundwork for uncovering complex correlations among microbiome composition, host gene expression and tumor characteristics.

A hypothesis-driven approach focuses on answering specific, theory-informed questions about tumor–microbe interactions. Researchers formulate these questions based on existing evidence or biological rationale and design experiments or analyses to test them directly. Key hypothesis-driven questions in the field include (1) what makes *F. nucleatum animalis* more efficient at invading colorectal tumors than other *F. nucleatum* strains; (2) what is the relative contribution of indole secretion and pathogen-associated molecular pattern recognition in the tumor response to *Lactobacillus reuteri*; (3) what are the unique mechanism and metabolic effects of *Helicobacter pylori* involved in gastric tumor invasion; and (4) can *Salmonella enterica* influence metastatic potential in breast tumor by altering epithelial–mesenchymal transition or extracellular matrix remodeling? Answering these questions is challenging, as the microbial composition within tumors is dynamic and evolves throughout tumor progression in different stages^90,91^. They cannot be fully resolved by metagenomic sequencing or 16S rRNA sequencing, rather by the integration of multimodel data^64^. Notably, the reported variations and inconsistencies of the presence of species between studies may greatly influence the validation and experimental reproducibility for downstream data-driven and hypothesis-driven analyses. Many microbes function as cooperative communities, in which their collective activities and interactions with the host proteome may be more relevant than the abundance of individual species. Therefore, a deeper understanding of how microbes alter signaling pathways and drive tumor progression requires well-designed in vitro and in vivo experiments.

## Best practices and recommendations

Identifying the tumor microbiome using bulk sequencing data holds great potential in understanding its biological and clinical significance. Best practices, decontaminated reference genome databases, rigorous computational pipelines and benchmarking practices are needed to reduce errors and derive new biological insights. Two major concerns should be considered during library preparation and sample sequencing. First, most library preparation reagents are designed to lyse host cell membranes and may not effectively disrupt microbial cell walls. This limitation can result in capturing only a small fraction of the microbial genome, particularly from extracellular microbes. Enhancing microbial cell lysis is crucial to reducing false negatives and increasing microbial read abundance. As standard techniques often fail to ensure effective reagent penetration into microbial cells, refining experimental protocols to achieve robust microbial lysis is essential for accurate tumor microbiome profiling^92^. Second, many library preparations involve the selection of human exons or poly(A) tails, which can reduce the probability of capturing microbial reads. To address these issues, the development of new sequencing technologies that can derive high-quality microbiome-targeted datasets from host samples is needed. Ideal pipelines should provide detailed step-by-step instructions, comprehensive parameter settings and optimization, and guidance on database selection to ensure proper usage and reproducibility. Furthermore, it is crucial to ensure comprehensive and community-level benchmarking of these pipelines (for example, the DREAM challenge), as there is no gold-standard method for profiling the microbiome from host sequencing data. Such benchmarking provides researchers with a straightforward comparison, facilitating informed decisions when selecting appropriate tools.

On the other hand, one could imagine that the integrative analysis of host bulk, single-cell and spatial transcriptomics theoretically can disentangle the effects of extracellular microbes from those of true tumor cell-penetrating microbes and enable us to quantify their true biological effect. As we continue to leverage genomic data in future studies, considering the inclusion of negative controls can greatly enhance our ability to accurately identify and analyze nonhuman DNA reads. Moreover, other omics data may also contribute to understanding host–microbiome interactions, such as microbially derived metabolites having opposite effects on tumor outcomes. For example, microbial metabolites in the pancreas activate macrophages^93^, whereas, in melanoma, CD8^+^ T cells are activated, leading to divergent effects on cancer control^44^. However, how to differentiate microbial-related metabolites from host metabolism is still challenging. Nevertheless, as mentioned before, emerging techniques, such as INVADE-seq, SHM-seq and MicroCART, incorporate microbe-targeted probe designs to enhance the success rate in capturing microbial reads^8,63,64^. We expect to see specific sequencing technologies being developed for the simultaneous measurement of multiomic signals from both host and tumor microbes in the near future. These exciting developments still require stringent study designs, starting from tissue collection and processing. The majority of tumor tissue samples continue to be preserved as formalin-fixed paraffin-embedded blocks. This involves multiple washing steps during tissue processing, which can result in the loss of the extracellular microbes that comprise the majority of the microbiome and limit much of the spatial detection of intracellular or highly abundant microbes^94^.

Although still in infancy, emerging evidence suggests that the tumor microbiome can affect host responses to anticancer therapies^95^ (Fig. 1b). Geller et al. demonstrated the role of Gammaproteobacteria in treatment with the chemotherapy drug gemcitabine in a colorectal cancer mouse model, in which antibiotic-treated mice exhibited a significant antitumor response to gemcitabine compared to control-treated mice, which showed rapid tumor progression^46^. Additionally, cultured bacteria from fresh human pancreatic ductal adenocarcinoma increased tumor resistance to gemcitabine in human colon carcinoma cell lines. Bacteria such as *F. nucleatum* can affect the host response to multiple therapeutics, including oxaliplatin, 5-fluorouracil and anti-PD-L1 antibodies, in mouse and preclinical models of colorectal cancer^96,97^. Overall, the tumor microbiome has the potential to influence therapy responses in cancer treatment. Many patients receive antibiotics before surgery or during treatment, potentially altering their tumor microbiome; hence, it is critical to consider clinical metadata while interpreting microbial profiles of patients with cancer. A deeper understanding of how the tumor microbiome interacts with antitumor therapies, along with comprehensive longitudinal clinical studies, is essential for advancing this field.

## Conclusion

Using human tumor sequencing data to study microbes presents a significant and challenging endeavor within the microbiome research community. In this Perspective, we reviewed evidence for the presence of microbes in tumor tissues and the difficulties in developing reliable methods and tools for this research. We emphasized the importance of investigating the tumor microbiome to understand fundamental biological interactions between microbes and tumor tissues. This understanding can elucidate microbial roles in tumor development, progression and response to treatments. We hope that our perspectives will support sustained efforts in the computational research community to study tumor microbiomes. We believe that mature protocols for contamination removal, advances in sequencing technologies and the development of suitable computational models and experimental validation will ultimately enhance our understanding of tumor biology.

## Figures and Tables

**Table 1 |:** Summary of critical issues that are challenging in tumor microbiome discovery and perspectives on potential best practices to overcome these issues

Steps	Critical issues	Best practices
**Data acquisition**	Raw sequence data are not open to the public	Use controlled access (best) or raw unaligned reads
	Low microbial read detection and technical depletion of microbes	Consider ribodepletion and minimize poly(A) enrichment Apply enzymatic treatment before lysis
**Reference mapping**	Contaminated reference genomes	Include human and contaminant ‘bait’ in reference Human masking in drafting microbial genomes
**Decontamination**	Non-tumor sources of low-abundance microbial reads	Include negative controls for sequencing
**Downstream analysis**	Count matrix has high sparsity and heterogeneity	Use larger sample size Apply deep learning methods
	False positives and negatives oriented from mapping step	Intersect multiple datasets
	Use of inappropriate normalization that induces noise	Preserve 0 values to reduce false positives
**Prediction validation**	Lacks matched data for crossvalidation	Integrative analysis of orthogonal methods and multiomics
	Lack of gold-standard data for validation	Design laboratory-controlled models and create gold-standard data	

**Table 2 |:** Bioinformatic approaches for deriving the tumor microbiome

Studies	Hoyd et al.^76^	Gihawi et al.^18^	Ghaddar et al.^86^	Lyu et al.^92^	Poore et al.^21^	Battaglia et al.^30^
**Data type**	Bulk RNA-seq	Bulk RNA-seq	scRNA-seq	ST	Bulk RNA-seq	WGS
**Alignment tools**						
Bowtie 2		✓			✓	
BWA						✓
Kraken2	✓		✓	✓		✓
KrakenUniq		✓			✓	
PathSeq						✓
BLAST				✓		
**Human reference genomes**						
Hg38	✓			✓		
Hg19		✓	✓		✓	✓
CHM13		✓			✓	✓
HPRC					✓	
**Microbial reference genomes**						
Kraken database					✓	
RefSeq			✓		✓	✓
WoLr1					✓	
MicrobialDB		✓				
Human-Associated Catalog						✓
**Reference genome cleaning**						
Conterminator					✓	
Exhaustive					✓	
**Filtering**						
Low taxon read removal	✓				✓	✓
Low unique *k*-mer removal			✓		✓	
Low genome coverage removal					✓	
Statistical false positive removal	✓		✓	✓		
Contaminant removal	✓	✓	✓			✓
**Batch correction**						
ConQuR					✓	
Voom-SNM	✓				✓	
LME						✓

HPRC, human pangenome; WoLr1, Web of Life database (10,575 genomes); ConQuR, conditional quantile regression; Voom-SNM, VOOM with supervised normalization method; LME, linear mixed effects models; ST, spatial transcriptomics. Analysis of microbiome data from host tumor sequencing typically involves multiple steps, including raw sequencing alignment, taxonomic classification, filtering and decontamination, and batch correction. The choice of bioinformatic approaches and tools for each step varies. For sequencing alignment, Kraken2 is a widely used, a *k*-mer-based taxonomic classification tool known for its speed, while KrakenUniq incorporates unique *k*-mer counting. PathSeq removes host reads, applies quality filtering before taxonomic classification and employs a read alignment-based approach like BWA. Common human reference genomes include Hg38 (GRCh38) and Hg19 (GRCh37), while CHM13 (T2T-CHM13) provides a complete genome, including previously missing regions and the Y chromosome. For microbial reference genomes, the Kraken database incorporates 59,963 microbial genomes from RepoPhlAn (14 June 2016), whereas RefSeq is a larger, publicly available database from the NCBI that includes viral, bacterial and archaeal genomes (for example, version 210 contains 29,648 genomes). MicrobialDB is a KrakenUniq-processed customized microbial database that includes only finished genomes of bacteria, viruses, eukaryotic pathogens and laboratory vectors. The Human-Associated Catalog (6,328 genomes) is a custom database derived from RefSeq genomes collected from human-associated microbial surveys. For contaminant removal, tools like Conterminator and Exhaustive clean microbial databases by removing mixed human reads. Filtering strategies depend on metrics such as taxon read counts, genome coverage and statistical- or literature-based false positive removal. For batch correction, methods such as ConQuR, Voom-SNM and LME are applied.

## BOX 1 Key terminology used in cancer microbiome research

Top left, microbiota refers to the community of microbes in a specific environment, including bacteria, viruses, fungi, archaea and protozoa. With high-throughput sequencing development, the microbiota and their genetic materials can be identified and quantified, leading to the term microbiome, which refers to the collective genomes of the microbiota. Bottom, the cancer microbiome broadly describes microbiomes associated with cancer, whether in the gut, solid tumors or hematologic malignancies. These immune-mediated interactions between tumors, immune cells and microbes in the TME are defined as the immuno-oncology microbiome. Focusing on solid tumors, both tumor microbiome and intratumoral microbiome refer to microbial genomes detected in tumors and adjacent normal tissues. Top right, the tumor-resident and/or tumor-colonizing microbe or microbiome emphasizes the microbiome of microbes that have adapted to the TME and established a stable presence. However, without observation of the microbiome for a long time (for example, longitudinal data), it remains difficult to determine whether the detected microbiome truly represents resident microbes or transient colonizers. By contrast, the tumor-infiltrating microbe or microbiome describes microbes that migrate into solid tumors from other sites, where they interact with the TME and exert functional effects.
